# 6-Bromo-1,3-bis­[(1,3-dioxolan-2-yl)meth­yl]-1*H*-imidazo[4,5-*b*]pyridin-2(3*H*)-one

**DOI:** 10.1107/S1600536813014591

**Published:** 2013-06-08

**Authors:** Youssef Kandri Rodi, Amal Haoudi, Frédéric Capet, Ahmed Mazzah, El Mokhtar Essassi, Lahcen El Ammari

**Affiliations:** a Laboratoire de Chimie Organique Appliquée, Université Sidi Mohamed, Ben Abdallah, Faculté des Sciences et Techniques, Route d’Immouzzer, BP 2202 Fès, Morocco; b Unité de Catalyse et de Chimie du Solide (UCCS), UMR 8181, Ecole Nationale Supérieure de Chimie de Lille, France; c USR 3290 Miniaturisation pour l’analyse, la synthèse et la protéomique, 59655 Villeneuve d’Ascq Cedex, Université Lille 1, France; d Laboratoire de Chimie Organique Hétérocyclique,URAC 21, Pôle de, compétences Pharmacochimie, Université Mohammed V-Agdal, BP 1014 Avenue Ibn Batouta, Rabat, Morocco; e Laboratoire de Chimie du Solide Appliquée, Faculté des Sciences, Université Mohammed V-Agdal, Avenue Ibn Battouta, BP 1014, Rabat, Morocco

## Abstract

In the title compound, C_14_H_16_BrN_3_O_5_, the N atoms adjacent to the carbonyl group in the five-membered ring are substituted by (1,3-dioxolan-2-yl)methyl groups. The fused ring system is essentially planar, with the largest deviation from the mean plane being 0.014 (2) Å for the C atom bearing the Br atom. The first oxolane ring, attached on the side of the N atom belonging to the pyridine ring, has an envelope conformation with one of the O atoms as the flap, whereas the second oxolane ring displays a twisted boat conformation. The two oxolane rings display envelope and twisted boat conformations. In the crystal, mol­ecules are linked by C—H⋯O hydrogen bonds, building chains parallel to the *a*-axis direction.

## Related literature

For the biological activity of imidazo­pyridine derivatives, see: Temple *et al.* (1987 ▶); Barraclough *et al.* (1990 ▶); Janssens *et al.* (1985 ▶); Liu *et al.* (2008 ▶); Bavetsias *et al.* (2010 ▶); Coates *et al.* (1993 ▶); For the chemistry of synthetic dyes, see: Ryabukhin *et al.* (2006 ▶); Schiffmann *et al.* (2006 ▶).
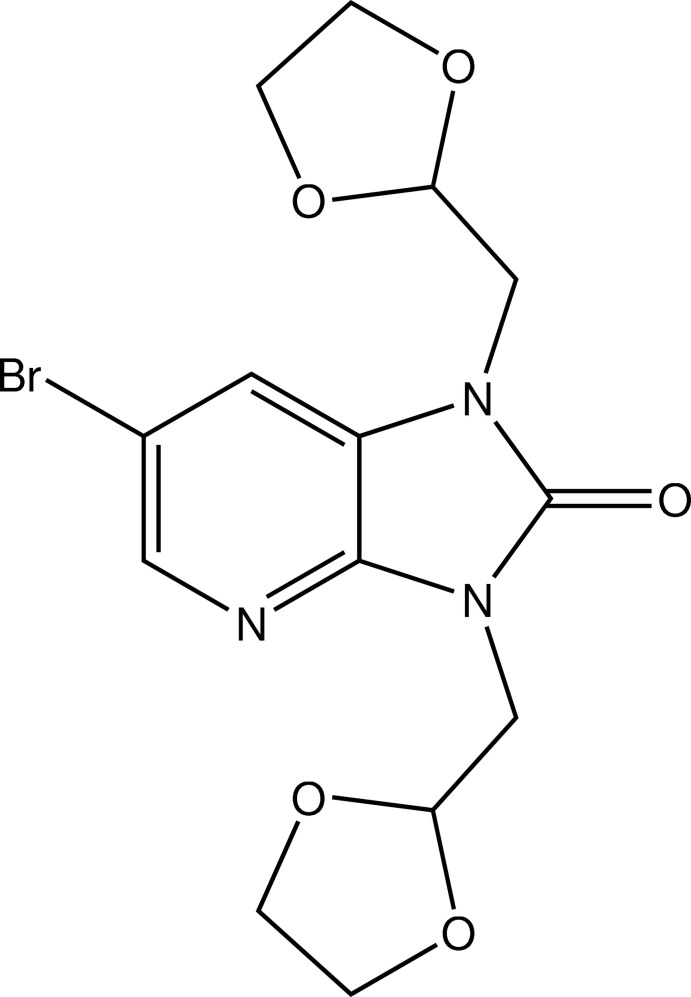


## Experimental

### 

#### Crystal data

C_14_H_16_BrN_3_O_5_
*M* *_r_* = 386.21
Monoclinic, 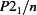

*a* = 5.1144 (1) Å
*b* = 17.8029 (4) Å
*c* = 16.5365 (5) Å
β = 97.009 (2)°
*V* = 1494.42 (6) Å^3^
*Z* = 4
Mo *K*α radiation
μ = 2.78 mm^−1^
*T* = 296 K
0.15 × 0.07 × 0.02 mm

#### Data collection

Bruker APEXII CCD diffractometer
Absorption correction: multi-scan (*SADABS*; Bruker, 2009 ▶) *T*_min_ = 0.674, *T*_max_ = 0.936
13392 measured reflections
3421 independent reflections
2632 reflections with *I* > 2σ(*I*)
*R*_int_ = 0.027

#### Refinement

*R*[*F* ^2^ > 2σ(*F* ^2^)] = 0.035
*wR*(*F* ^2^) = 0.089
*S* = 1.02
3421 reflections
208 parameters
H-atom parameters constrained
Δρ_max_ = 0.45 e Å^−3^
Δρ_min_ = −0.27 e Å^−3^

### 

Data collection: *APEX2* (Bruker, 2009 ▶); cell refinement: *APEX2*; data reduction: *SAINT* (Bruker, 2009 ▶); program(s) used to solve structure: *SHELXS97* (Sheldrick, 2008 ▶); program(s) used to refine structure: *SHELXL97* (Sheldrick, 2008 ▶); molecular graphics: *ORTEP-3 for Windows* (Farrugia, 2012 ▶); software used to prepare material for publication: *PLATON* (Spek, 2009 ▶) and *publCIF* (Westrip, 2010 ▶).

## Supplementary Material

Crystal structure: contains datablock(s) I, global. DOI: 10.1107/S1600536813014591/fj2631sup1.cif

Structure factors: contains datablock(s) I. DOI: 10.1107/S1600536813014591/fj2631Isup2.hkl

Supplementary material file. DOI: 10.1107/S1600536813014591/fj2631Isup3.cml

Additional supplementary materials:  crystallographic information; 3D view; checkCIF report

## Figures and Tables

**Table 1:** Hydrogen-bond geometry (Å, °)

*D*—H⋯*A*	*D*—H	H⋯*A*	*D*⋯*A*	*D*—H⋯*A*
C10—H10*B*⋯O2^i^	0.97	2.36	3.291 (4)	160

Symmetry code: (i) 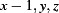.

**Fig. 1.**
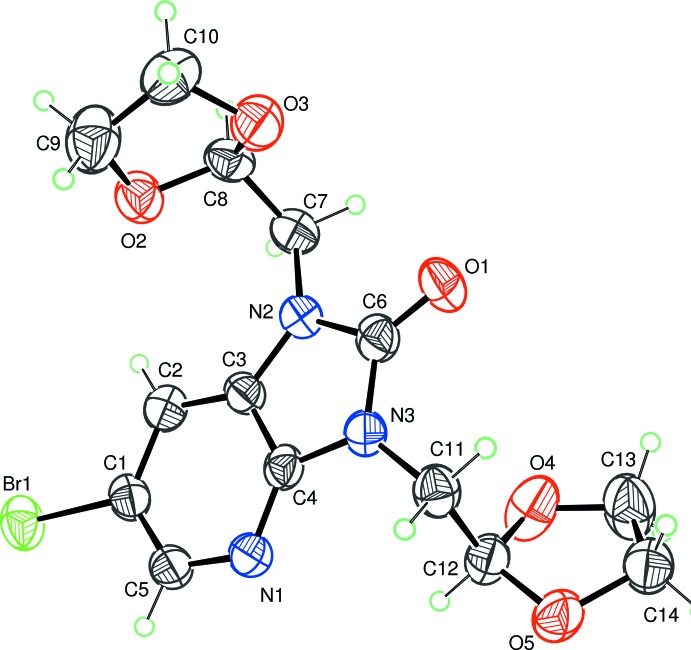
: Molecular plot the title compound with the atom-labelling scheme. Displacement ellipsoids are drawn at the 50% probability level. H atoms are represented as small circles.

**Fig. 2.**
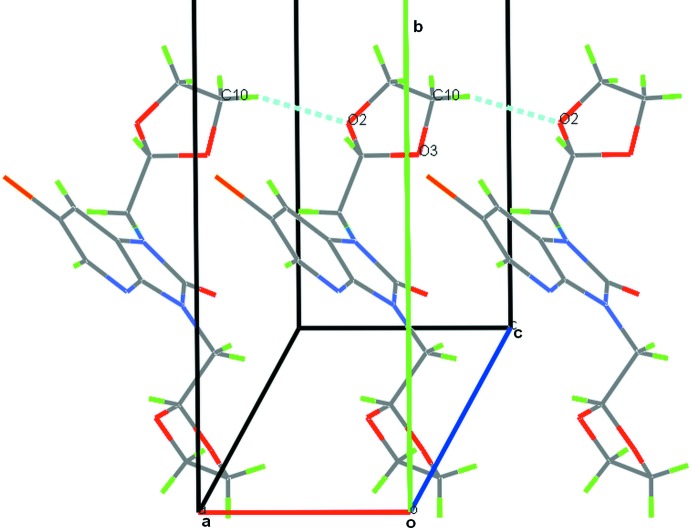
: Intermolecular interactions in the title compound building a a chain parallel to a axis. Hydrogen bonds are shown as dashed lines.

## supplementary crystallographic information

### Comment

Substituted imidazopyridines and structurally related compounds are of pharmacological and therapeutical interest. They have been tested for their potential as anticancer (Temple *et al.*, 1987), inotropic (Barraclough *et al.*, 1990), selective antihistamine (H1) agents (Janssens *et al.*, 1985) and antibacterial activity (Liu *et al.*, 2008). Imidazo[4,5-*b*]pyridine derivatives were also reported as Aurora kinases (Bavetsias *et al.*, 2010), and cyclic PDE inhibitors (Coates *et al.*, 1993).The preparation of these compounds is usually straightforward, and a number of synthetic methods are already available (Ryabukhin *et al.*, 2006; Schiffmann *et al.*, 2006). In this letter, we report the synthesis of 1,3-bis(methyl-1,3-dioxolane)-6- bromo-1,3-dihydroimidazo[4,5-*b*]pyridin-2-one *via* the reaction between 6- bromo-1,3-dihydro-imidazo[4,5-*b*]pyridin-2-one and 2-chloromethyl-1,3- dioxolane in DMF using K_2_CO_3_ as base (scheme 1).

The molecule of title compound, 3-benzyl-6-bromo-1,3-dihydro-imidazo [4,5-*b*]pyridin-2-one, build up from two fused five- and six-membered rings liked on opposite sides to (1,3-dioxolan-2-yl)methyl groups as shown in Fig. 1. The fused rings system (N1N2N3 C1 to C6) is essentially planar with the largest deviation from the mean plane being 0.014 (2) A° at C1 atom. The five-membered ring (O2O3C8C9C10) adopts on C8–O3 a twisted conformation, whereas the other (O4O5C12C13C14) an envelope conformation (on O5), as indicated by the total puckering amplitudes Q2 = 0.355 (3) Å; and Q2 = 0.234 (3) Å and spherical polar angles φ2 = 238.6 (5)° and φ2 = 65.1 (7)°, respectively. The dihedral angles between the fused imidazole and pyridine rings and the two oxolane cycles (O2O3C8C9C10) and (O4O5C12C13C14) are of 82.34 (13) ° and 38.15 (13) °, respectively.

In the crystal, the molecules are linked by C10–H10···O2 hydrogen bond in the way to build a chain parallel to *a* axis as shown in Fig. 2 and Table 2.

### Experimental

To a stirred solution of 6-bromo-1,3-dihydro-imidazo[4,5-*b*]pyridin-2-one (0.5 g; 2.33 mmol), K_2_CO_3_ (1.29 g; 9.34 mmol), and tetra n-butylammonium bromide (0.07 g; 2.37 10 ^-4^ mol) in DMF, 2-chloromethyl-1,3-dioxolane (5.84 mmol) was added dropwise. Stirring was continued at room temperature for 24 h. After completion of reaction (monitored by TLC), the salt was filtered and the solvent was removed under reduced pressure. The resulting residue was purified by column chromatography on silica gel using (ethylacetate/hexane (1/1) as eluent. The yield of the reaction is of 73%. Crystals were isolated after the solvent (hexane/acetate d'ethyle: 3/2) was allowed to evaporate.

### Refinement

All H atoms could be located in a difference Fourier map. However, they were placed in calculated positions with C—H = 0.93 Å (aromatic), N—H = 0.86 and C—H = 0.97 Å (methylene) and refined as riding on their parent atoms with *U*_iso_(H) = 1.2 *U*_eq_ (C, N).

### Crystal data

**Table:**

C_14_H_16_BrN_3_O_5_	*F*(000) = 784
*M**_r_* = 386.21	*D*_x_ = 1.717 Mg m^−^^3^
Monoclinic, *P*2_1_/*n*	Melting point: 446 K
Hall symbol: -P 2yn	Mo *K*α radiation, λ = 0.71073 Å
*a* = 5.1144 (1) Å	Cell parameters from 4379 reflections
*b* = 17.8029 (4) Å	θ = 2.3–25.5°
*c* = 16.5365 (5) Å	µ = 2.78 mm^−^^1^
β = 97.009 (2)°	*T* = 296 K
*V* = 1494.42 (6) Å^3^	Needle, white
*Z* = 4	0.15 × 0.07 × 0.02 mm

### Data collection

**Table:**

Bruker APEXII CCD diffractometer	3421 independent reflections
Radiation source: microfocus source	2632 reflections with *I* > 2σ(*I*)
Graphite monochromator	*R*_int_ = 0.027
φ and ω scans	θ_max_ = 27.5°, θ_min_ = 1.7°
Absorption correction: multi-scan (*SADABS*; Bruker, 2009)	*h* = −6→5
*T*_min_ = 0.674, *T*_max_ = 0.936	*k* = −23→23
13392 measured reflections	*l* = −21→20

### Refinement

**Table:**

Refinement on *F*^2^	Primary atom site location: structure-invariant direct methods
Least-squares matrix: full	Secondary atom site location: difference Fourier map
*R*[*F*^2^ > 2σ(*F*^2^)] = 0.035	Hydrogen site location: difference Fourier map
*wR*(*F*^2^) = 0.089	H-atom parameters constrained
*S* = 1.02	*w* = 1/[σ^2^(*F*_o_^2^) + (0.0418*P*)^2^ + 0.7351*P*] where *P* = (*F*_o_^2^ + 2*F*_c_^2^)/3
3421 reflections	(Δ/σ)_max_ = 0.010
208 parameters	Δρ_max_ = 0.45 e Å^−^^3^
0 restraints	Δρ_min_ = −0.27 e Å^−^^3^

### Special details

**Table:**

Geometry. All s.u.'s (except the s.u. in the dihedral angle between two l.s. planes) are estimated using the full covariance matrix. The cell s.u.'s are taken into account individually in the estimation of s.u.'s in distances, angles and torsion angles; correlations between s.u.'s in cell parameters are only used when they are defined by crystal symmetry. An approximate (isotropic) treatment of cell s.u.'s is used for estimating s.u.'s involving l.s. planes.
Refinement. Refinement of *F*^2^ against all reflections. The weighted *R*-factor *wR* and goodness of fit *S* are based on *F*^2^, conventional *R*-factors *R* are based on *F*, with *F* set to zero for negative *F*^2^. The threshold expression of *F*^2^ > σ(*F*^2^) is used only for calculating *R*-factors(gt) *etc*. and is not relevant to the choice of reflections for refinement. *R*-factors based on *F*^2^ are statistically about twice as large as those based on *F*, and *R*- factors based on all data will be even larger.

### Fractional atomic coordinates and isotropic or equivalent isotropic displacement parameters (Å^2^)

**Table:**

	*x*	*y*	*z*	*U*_iso_*/*U*_eq_	
Br1	1.05083 (6)	0.395344 (15)	0.301591 (18)	0.05380 (12)	
C1	0.8031 (5)	0.32618 (13)	0.33438 (15)	0.0401 (5)	
C2	0.7746 (5)	0.32059 (13)	0.41724 (15)	0.0412 (5)	
H2	0.8760	0.3485	0.4570	0.049*	
C3	0.5856 (5)	0.27082 (12)	0.43522 (14)	0.0371 (5)	
C4	0.4416 (5)	0.23061 (12)	0.37221 (14)	0.0360 (5)	
C5	0.6557 (5)	0.28362 (13)	0.27657 (15)	0.0419 (6)	
H5	0.6855	0.2890	0.2225	0.050*	
C6	0.2946 (5)	0.19654 (13)	0.49010 (15)	0.0411 (6)	
C7	0.6085 (5)	0.26864 (15)	0.58864 (15)	0.0465 (6)	
H7A	0.7984	0.2710	0.5898	0.056*	
H7B	0.5692	0.2289	0.6254	0.056*	
C8	0.5135 (5)	0.34154 (15)	0.61902 (15)	0.0444 (6)	
H8	0.6117	0.3526	0.6723	0.053*	
C9	0.3556 (7)	0.45586 (17)	0.5782 (2)	0.0689 (9)	
H9A	0.2573	0.4709	0.5269	0.083*	
H9B	0.4373	0.5000	0.6049	0.083*	
C10	0.1799 (6)	0.41836 (17)	0.6312 (2)	0.0583 (7)	
H10A	0.2136	0.4365	0.6868	0.070*	
H10B	−0.0039	0.4269	0.6111	0.070*	
C11	0.0926 (5)	0.13044 (14)	0.36364 (16)	0.0432 (6)	
H11A	−0.0073	0.1535	0.3165	0.052*	
H11B	−0.0310	0.1123	0.3992	0.052*	
C12	0.2506 (5)	0.06459 (13)	0.33618 (16)	0.0444 (6)	
H12	0.3725	0.0829	0.2993	0.053*	
C13	0.2579 (7)	−0.03685 (19)	0.4183 (2)	0.0718 (10)	
H13A	0.1987	−0.0342	0.4718	0.086*	
H13B	0.3727	−0.0801	0.4169	0.086*	
C14	0.0312 (6)	−0.04352 (16)	0.35507 (17)	0.0545 (7)	
H14A	0.0209	−0.0936	0.3318	0.065*	
H14B	−0.1320	−0.0327	0.3771	0.065*	
N1	0.4701 (4)	0.23447 (11)	0.29392 (12)	0.0411 (5)	
N2	0.4931 (4)	0.24938 (11)	0.50679 (12)	0.0406 (5)	
N3	0.2652 (4)	0.18604 (10)	0.40653 (12)	0.0395 (5)	
O1	0.1709 (4)	0.16624 (10)	0.53895 (11)	0.0539 (5)	
O2	0.5490 (4)	0.40116 (10)	0.56540 (12)	0.0518 (5)	
O3	0.2459 (4)	0.34071 (11)	0.62657 (12)	0.0572 (5)	
O4	0.3926 (4)	0.02910 (10)	0.40233 (13)	0.0639 (6)	
O5	0.0812 (4)	0.01107 (10)	0.29556 (11)	0.0549 (5)	

### Atomic displacement parameters (Å^2^)

**Table:**

	*U*^11^	*U*^22^	*U*^33^	*U*^12^	*U*^13^	*U*^23^
Br1	0.04950 (18)	0.04746 (16)	0.0682 (2)	−0.00250 (12)	0.02211 (14)	0.00902 (13)
C1	0.0395 (13)	0.0352 (12)	0.0480 (14)	0.0052 (10)	0.0156 (11)	0.0052 (10)
C2	0.0402 (13)	0.0381 (12)	0.0456 (14)	−0.0001 (10)	0.0061 (11)	−0.0009 (10)
C3	0.0411 (13)	0.0345 (11)	0.0371 (12)	0.0033 (10)	0.0110 (10)	0.0028 (9)
C4	0.0382 (13)	0.0285 (10)	0.0421 (13)	0.0040 (9)	0.0083 (10)	0.0012 (9)
C5	0.0507 (15)	0.0361 (12)	0.0413 (13)	0.0077 (11)	0.0154 (11)	0.0031 (10)
C6	0.0449 (14)	0.0343 (12)	0.0451 (14)	0.0067 (10)	0.0096 (11)	0.0049 (10)
C7	0.0487 (15)	0.0517 (14)	0.0390 (13)	0.0017 (12)	0.0047 (11)	0.0042 (11)
C8	0.0449 (15)	0.0550 (15)	0.0328 (13)	−0.0039 (12)	0.0031 (11)	−0.0012 (11)
C9	0.063 (2)	0.0536 (17)	0.092 (2)	0.0057 (15)	0.0180 (18)	0.0063 (16)
C10	0.0472 (16)	0.0575 (16)	0.071 (2)	−0.0025 (13)	0.0103 (14)	−0.0145 (15)
C11	0.0407 (14)	0.0414 (12)	0.0466 (14)	−0.0022 (11)	0.0016 (11)	0.0035 (11)
C12	0.0479 (15)	0.0362 (12)	0.0474 (14)	−0.0016 (11)	−0.0010 (12)	0.0002 (11)
C13	0.080 (2)	0.065 (2)	0.066 (2)	−0.0116 (17)	−0.0120 (18)	0.0195 (16)
C14	0.0622 (18)	0.0474 (15)	0.0531 (17)	−0.0086 (13)	0.0031 (14)	0.0015 (12)
N1	0.0492 (12)	0.0361 (10)	0.0391 (11)	0.0042 (9)	0.0094 (9)	0.0013 (8)
N2	0.0464 (12)	0.0389 (10)	0.0377 (11)	−0.0022 (9)	0.0093 (9)	0.0011 (9)
N3	0.0444 (12)	0.0328 (10)	0.0428 (11)	−0.0015 (9)	0.0110 (9)	0.0006 (8)
O1	0.0597 (12)	0.0524 (11)	0.0531 (11)	−0.0068 (9)	0.0206 (9)	0.0100 (9)
O2	0.0510 (11)	0.0498 (10)	0.0568 (11)	−0.0021 (8)	0.0158 (9)	0.0048 (9)
O3	0.0526 (12)	0.0514 (11)	0.0719 (13)	−0.0062 (9)	0.0248 (10)	−0.0048 (9)
O4	0.0615 (13)	0.0453 (10)	0.0764 (14)	0.0050 (9)	−0.0258 (11)	0.0006 (10)
O5	0.0716 (14)	0.0457 (10)	0.0431 (10)	−0.0086 (9)	−0.0105 (9)	−0.0016 (8)

### Geometric parameters (Å, º)

**Table:**

Br1—C1	1.893 (2)	C9—O2	1.422 (4)
C1—C5	1.371 (4)	C9—C10	1.488 (4)
C1—C2	1.399 (3)	C9—H9A	0.9700
C2—C3	1.370 (3)	C9—H9B	0.9700
C2—H2	0.9300	C10—O3	1.427 (3)
C3—N2	1.381 (3)	C10—H10A	0.9700
C3—C4	1.398 (3)	C10—H10B	0.9700
C4—N1	1.322 (3)	C11—N3	1.452 (3)
C4—N3	1.375 (3)	C11—C12	1.524 (4)
C5—N1	1.347 (3)	C11—H11A	0.9700
C5—H5	0.9300	C11—H11B	0.9700
C6—O1	1.212 (3)	C12—O4	1.389 (3)
C6—N3	1.385 (3)	C12—O5	1.403 (3)
C6—N2	1.387 (3)	C12—H12	0.9800
C7—N2	1.450 (3)	C13—O4	1.403 (4)
C7—C8	1.494 (4)	C13—C14	1.469 (4)
C7—H7A	0.9700	C13—H13A	0.9700
C7—H7B	0.9700	C13—H13B	0.9700
C8—O3	1.389 (3)	C14—O5	1.428 (3)
C8—O2	1.409 (3)	C14—H14A	0.9700
C8—H8	0.9800	C14—H14B	0.9700
			
C5—C1—C2	121.9 (2)	O3—C10—H10B	111.0
C5—C1—Br1	119.34 (18)	C9—C10—H10B	111.0
C2—C1—Br1	118.71 (19)	H10A—C10—H10B	109.0
C3—C2—C1	114.8 (2)	N3—C11—C12	110.9 (2)
C3—C2—H2	122.6	N3—C11—H11A	109.5
C1—C2—H2	122.6	C12—C11—H11A	109.5
C2—C3—N2	133.6 (2)	N3—C11—H11B	109.5
C2—C3—C4	119.4 (2)	C12—C11—H11B	109.5
N2—C3—C4	107.0 (2)	H11A—C11—H11B	108.0
N1—C4—N3	126.2 (2)	O4—C12—O5	107.53 (19)
N1—C4—C3	126.3 (2)	O4—C12—C11	111.1 (2)
N3—C4—C3	107.5 (2)	O5—C12—C11	110.3 (2)
N1—C5—C1	123.5 (2)	O4—C12—H12	109.3
N1—C5—H5	118.2	O5—C12—H12	109.3
C1—C5—H5	118.2	C11—C12—H12	109.3
O1—C6—N3	127.1 (2)	O4—C13—C14	107.2 (2)
O1—C6—N2	126.8 (2)	O4—C13—H13A	110.3
N3—C6—N2	106.1 (2)	C14—C13—H13A	110.3
N2—C7—C8	114.0 (2)	O4—C13—H13B	110.3
N2—C7—H7A	108.7	C14—C13—H13B	110.3
C8—C7—H7A	108.7	H13A—C13—H13B	108.5
N2—C7—H7B	108.7	O5—C14—C13	104.1 (2)
C8—C7—H7B	108.7	O5—C14—H14A	110.9
H7A—C7—H7B	107.6	C13—C14—H14A	110.9
O3—C8—O2	105.6 (2)	O5—C14—H14B	110.9
O3—C8—C7	112.7 (2)	C13—C14—H14B	110.9
O2—C8—C7	111.7 (2)	H14A—C14—H14B	109.0
O3—C8—H8	108.9	C4—N1—C5	114.0 (2)
O2—C8—H8	108.9	C3—N2—C6	109.6 (2)
C7—C8—H8	108.9	C3—N2—C7	126.3 (2)
O2—C9—C10	105.2 (2)	C6—N2—C7	123.4 (2)
O2—C9—H9A	110.7	C4—N3—C6	109.7 (2)
C10—C9—H9A	110.7	C4—N3—C11	125.6 (2)
O2—C9—H9B	110.7	C6—N3—C11	124.4 (2)
C10—C9—H9B	110.7	C8—O2—C9	106.0 (2)
H9A—C9—H9B	108.8	C8—O3—C10	103.7 (2)
O3—C10—C9	103.8 (2)	C12—O4—C13	107.9 (2)
O3—C10—H10A	111.0	C12—O5—C14	106.51 (19)
C9—C10—H10A	111.0		
			
C5—C1—C2—C3	−1.3 (3)	N3—C6—N2—C7	−171.4 (2)
Br1—C1—C2—C3	178.13 (17)	C8—C7—N2—C3	87.4 (3)
C1—C2—C3—N2	−179.0 (2)	C8—C7—N2—C6	−103.0 (3)
C1—C2—C3—C4	0.3 (3)	N1—C4—N3—C6	179.4 (2)
C2—C3—C4—N1	1.0 (4)	C3—C4—N3—C6	−0.4 (3)
N2—C3—C4—N1	−179.6 (2)	N1—C4—N3—C11	5.4 (4)
C2—C3—C4—N3	−179.2 (2)	C3—C4—N3—C11	−174.4 (2)
N2—C3—C4—N3	0.2 (3)	O1—C6—N3—C4	179.9 (2)
C2—C1—C5—N1	1.3 (4)	N2—C6—N3—C4	0.4 (3)
Br1—C1—C5—N1	−178.18 (18)	O1—C6—N3—C11	−6.0 (4)
N2—C7—C8—O3	64.1 (3)	N2—C6—N3—C11	174.6 (2)
N2—C7—C8—O2	−54.6 (3)	C12—C11—N3—C4	68.0 (3)
O2—C9—C10—O3	−14.6 (3)	C12—C11—N3—C6	−105.2 (3)
N3—C11—C12—O4	60.3 (3)	O3—C8—O2—C9	31.3 (3)
N3—C11—C12—O5	179.4 (2)	C7—C8—O2—C9	154.1 (2)
O4—C13—C14—O5	12.2 (4)	C10—C9—O2—C8	−9.6 (3)
N3—C4—N1—C5	179.2 (2)	O2—C8—O3—C10	−40.7 (3)
C3—C4—N1—C5	−1.1 (3)	C7—C8—O3—C10	−162.9 (2)
C1—C5—N1—C4	0.0 (3)	C9—C10—O3—C8	33.5 (3)
C2—C3—N2—C6	179.4 (3)	O5—C12—O4—C13	−18.7 (3)
C4—C3—N2—C6	0.1 (3)	C11—C12—O4—C13	102.1 (3)
C2—C3—N2—C7	−9.8 (4)	C14—C13—O4—C12	3.6 (4)
C4—C3—N2—C7	170.9 (2)	O4—C12—O5—C14	26.6 (3)
O1—C6—N2—C3	−179.8 (2)	C11—C12—O5—C14	−94.7 (2)
N3—C6—N2—C3	−0.3 (3)	C13—C14—O5—C12	−23.4 (3)
O1—C6—N2—C7	9.1 (4)		

### Hydrogen-bond geometry (Å, º)

**Table:**

*D*—H···*A*	*D*—H	H···*A*	*D*···*A*	*D*—H···*A*
C10—H10*B*···O2^i^	0.97	2.36	3.291 (4)	160

Symmetry code: (i) *x*−1, *y*, *z*.
